# Strengthening channeling policy: the Finnish approach to protecting domestic online gambling market

**DOI:** 10.3389/fsoc.2023.1272735

**Published:** 2024-01-11

**Authors:** Johanna Järvinen-Tassopoulos, Virve Marionneau, Michael Egerer

**Affiliations:** ^1^Department of Public Health and Welfare, Finnish Institute for Health and Welfare, Helsinki, Finland; ^2^Faculty of Social Sciences, Centre for Research on Addiction, Control, and Governance, University of Helsinki, Helsinki, Finland

**Keywords:** offshore gambling, channeling, gambling regime, policy, regulatory models, Finland

## Abstract

Many jurisdictions struggle to curb offshore gambling as established approaches to gambling regulation no longer apply to online gambling. This study aimed to examine the arguments used by Finnish stakeholders who discussed channeling as a tool to curb offshore gambling and the monopolistic gambling regime as a sustainable framework to tackle the issue of offshore gambling. In total, 18 Finnish public servants employed in ministries in charge of gambling matters and representatives of Veikkaus, the state-owned gambling company, were interviewed for the purpose of this study. Channeling was described by the interviewees as an important policy tool but without a regulatory framework to block offshore operation, it would remain an ideal type of political strategy. Beside the monopolistic gambling regime, the pros and cons of a potential licensing regime were discussed. This study showed that legislative and regulatory changes form a lengthy political process; the decrease of the market share of online gambling marked the beginning of a new era in Finland’s gambling policy.

## 1 Introduction

Many jurisdictions struggle to curb offshore gambling. Offshore gambling originates from across national or jurisdictional border, and this makes the online gambling sector difficult to regulate with jurisdiction specific structures (Gainsbury et al., 2018a). As the online marketplace is highly competitive, regulatory policies in different jurisdictions struggle to keep pace with the technological development of the online gambling industry (also Banks, 2014; Järvinen-Tassopoulos, 2022). In more restrictive jurisdictions, including those operating a monopoly system for gambling (e.g., Norway, Finland, most Canadian provinces), a strategic tool called channeling has become increasingly important. Channeling refers to national authorities attempting to direct online gambling toward the regulated market with different measures. Channeling can be accomplished by making the regulated offer more appealing to consumers (e.g., offering attractive gambling products, higher return rates, and more intensive marketing). Conversely, channeling can be also accomplished by making unregulated offer less appealing for consumers. Measures to accomplish this may range from criminalizing playing on offshore gambling sites to softer measures (e.g., limiting or banning advertising) (Lycka, 2014; Hörnle et al., 2018; Egerer and Marionneau, 2023). National and state-owned gambling companies are more dependent of the jurisdiction’s channeling policy, as they cannot compete with the private gambling companies with a multitude of attractive games, welcoming bonuses, and intensive marketing (Nadeau et al., 2014; Littler and Järvinen-Tassopoulos, 2018; Borch, 2022).

This study scrutinizes channeling as a tool to curb offshore provision of gambling in a monopolistic regime. Previously, only a few studies (e.g., Nadeau et al., 2014; Littler and Järvinen-Tassopoulos, 2018; Borch, 2022; Selkee et al., 2022) have explored the operative and political consequences of the channeling tool. However, to our knowledge, no previous studies have explored the experiences, views, and challenges of channeling from the perspective of representatives of the regulators and gambling providers. The study is situated within the context of Finland, a Nordic Member State of the EU.

In June 2023, the recently elected right-wing Finnish government decided to reregulate the national online gambling market by introducing licenses to outside providers as of 2026. The move follows developments in most other European countries where gambling monopolies have already been dissolved and online markets have been opened to competition. The reasoning behind this decision was based particularly on the difficulty to control the offshore market and to channel consumption to the monopoly, despite attempts to strengthen the monopoly (Rydman and Tukia, 2019). In 2021, only an estimated 65 percent of the Finnish online gambling market was controlled by the monopoly (H2 Gambling Capital, 2021), up from 14 percent in 2019 (Veikkaus, 2018). According to different estimates, Finns lose between 300 million and 470 million euros to offshore websites annually (Sailas et al., 2023).

In total 18 public servants employed in different Finnish Ministries in charge of gambling related matters and representatives of the Finnish state-owned gambling company (Veikkaus) were interviewed for the purpose of this study between winter 2018 and spring 2019. During the time of the interviews, channeling was one of the cornerstones of the Finnish gambling policy, along with the prevention of gambling harms. However, curbing offshore gambling had proven to be difficult to achieve, even though the previous government of Prime Minister Sanna Marin outlined that Veikkaus should be able evolve in the changing online environment and improve its operation in a responsible manner (Rydman and Tukia, 2019).

Our study focuses on the thoughts and arguments of the Finnish stakeholders concerning the implementation of channeling measures in order to reduce offshore gambling and on the socio-economic consequences of the loss of the online market share. Our research questions are the following: (1) Is channeling considered an effective tool to curb offshore gambling offer? and (2) Is maintaining a monopolistic regime considered a sustainable solution to tackle the issue of offshore gambling?

## 2 Online gambling regulation within the European single market

What makes the regulation of online gambling especially challenging in the European context is the fact that some of the jurisdictions (e.g., Malta, Gibraltar, UK) serve as ‘online gambling hubs’, which issue gambling licenses to online operators (Zborowska et al., 2012; Myllymaa, 2017). It may be difficult for online gamblers to distinguish licensed operators from unlicensed ones when many of them offer gambling products and services in their native language (Gainsbury et al., 2018b). Thus, established approaches to gambling regulation no longer apply to online gambling and the prevention of gambling harms needs new, transnational measures (also Zborowska et al., 2012). Furthermore, most offshore gambling companies operate in the markets of the most harmful gambling products, such as online casino and online sports betting (see Gainsbury et al., 2018b).

Although the offshore gambling market is global, regulations are national. Even within the European Union (EU), gambling legislation has not been harmonized across Member States. On the contrary, the European Commission (EC) has an interest in safeguarding the EU treaties and in ensuring conformity across Member States (e.g., Littler, 2011). Gambling activities fall within the scope of article 49 of the Treaty of the Functioning of the EU (TFEU) on the freedom of services. This has limited the possibilities of Member States to restrict the fundamental freedom of providing services within the internal market. Any limitations need to be justifiable and proportional (see Miettinen, 2022). The regulatory reach of Member States regarding offshore gambling is therefore limited. In most of the Member States, the difficulties in justifying restrictions within the internal market have led to the dismantlement of monopolistic structures and their replacement with licensing regimes that allow operators from other Member States (often with a subsidiary in the country in question) to apply for an authorization to provide online gambling (e.g., Costes et al., 2016; Schmidt-Kessen et al., 2019). Malta is the key EU Member State that facilitate the establishment of private offshore companies by allowing them to operate online gambling from its jurisdiction (Zborowska et al., 2012; Myllymaa, 2017).

In addition to regulatory challenges, offshore gambling offer decreases gambling revenues across jurisdictions, as offshore companies do not usually pay tax in the jurisdictions they target (Gainsbury et al., 2018b; Borch, 2022; Järvinen-Tassopoulos, 2022). The global online market has grown over 100 percent since 2016 (H2 Gambling Capital, 2021). In 2022, the size of the global online gambling market was estimated at USD 75.2 billion, and it is expected to reach USD 217 billion by the end of 2031 (Growth Marketing Report, 2023).

Some measures are available for regulators to curb offshore provision. These measures include, e.g., blacklisting offshore websites, blocking IP addresses and monetary transactions to these sites, informing consumers, and charging offshore providers with fines and sanctions (Hörnle et al., 2018; Gainsbury et al., 2018a,b; Egerer and Marionneau, 2023). While in most contexts these measures have only been modestly effective, they are widely employed to advance the channeling objective. Channeling has therefore become a kind of a placeholder (Planzer, 2014) with varying contents depending on the interests of different stakeholders (*cf.* Selkee et al., 2022).

Previous research has shown that channeling policies are justified with at least three different rationales (*cf.* Van Den Bogaert and Cuyvers, 2011; Borch, 2022; Selkee et al., 2022): First, regulated markets are expected to have higher levels of consumer protection, making channeling a tool for gambling harm prevention. This of course presupposes that regulated gambling provision is indeed safer. Otherwise, increasing the size of the regulated market may result in more overall harms (*cf.* Nadeau et al., 2014). Second, regulated markets are expected to have fewer criminal activities (e.g., money laundering, fraud), making channeling also as a tool for legal enforcement. This objective is independent of the size of the regulated market. Then again, channeling is only justified in this regard, if the regulated offers are indeed sufficiently monitored. After all, legalized and licensed online gambling markets are also not immune to illegal activity or illegal competition (e.g., Spapens et al., 2008; Banks, 2017; Rolando and Scavarda, 2018). Third, gambling generates surplus of profits, which have traditionally directed to supplement national budgets and other beneficiaries (*cf.* Sulkunen et al., 2022). Offshore gambling challenges the premise of gambling as a means of revenue collection. Channeling can therefore also function as a tool for safeguarding public revenue. While not compatible with EU law, this objective may be behind many attempts to curb offshore gambling (e.g., Selin, 2019).

## 3 Materials and methods

### 3.1 Qualitative data

Eighteen thematic interviews were collected among public servants employed in Finnish Ministries in charge of gambling regulation, ownership steering, and prevention of gambling harms, and among representatives of Veikkaus employed in the executive team, in the board of directors, or in the administrative board. The interviews were conducted during winter 2018 and spring 2019. The potential interviewees were contacted by e-mail or by phone. Those who agreed to be interviewed could opt to receive the thematic questions in advance and had the right to decline to participate in the study at any time.

As the interviewees represented key stakeholders (and some of them the political elite) on the Finnish gambling scene, they can also be defined as “experts,” who “possess special knowledge of a social phenomenon which the interviewer is interested in” (Gläser and Laudel, 2009, p. 117). In all interviews, interests, trusts, power, control, and hierarchy can influence this form of social interaction (Abels and Behrens, 2009). Most of interviewees showed goodwill and provided the interviewers with important information on the current situation in the Finnish online gambling market. In a few cases, the conversational interaction was hindered by the ‘profile-effect’, which means that the interviewee sought to ‘show off’ in front of the interviewer (Abels and Behrens, 2009, p. 144). On the other hand, the principal researcher felt (JJT) pressured to gather information on the key stakeholders that would have an impact on the ownership steering of Veikkaus. In summary, the topics of ownership steering and online gambling operation were hot political issues when the interviews were conducted and they still are in Finland.

Two sets of questions were used to conduct the interviews. The public servants were interviewed on themes focusing on ownership steering, prevention of gambling harms, responsible gambling, and cooperation between Ministries and the Prime Minister’s Office (home of the Ownership Steering Office). The representatives of Veikkaus were interviewed using themes concerning operative strategy, ownership steering, online competition between licensed and unlicensed operators, responsible gambling, and the prevention of gambling harms.

The interviews were conducted in-person (except one by phone) and they lasted from 40 min to two hours. The interviews were transcribed verbatim. Additionally, the interviewees were guaranteed anonymity to protect their identity and they could ask to see their transcribed interview in case they wanted to change something in it. The excerpts from the interviews conducted among the representatives of Veikkaus are indicated with ‘VE’ and those from the interviews conducted among the public servants are indicated with ‘PS’. The identifying number after ‘VE’ or ‘PS’ indicates the numerical order in which the interviews were saved in the Atlas.ti software.

### 3.2 Qualitative method

The analysis of the interview data was conducted using the inductive content analysis as a qualitative method (Elo and Kyngäs, 2008). First, all the text material was read to identify passages related to ‘channeling’, ‘blocking’ and ‘gambling regime’ (Open Coding). Then, the different codes created based on the interviews were grouped and given specific titles (Grouping and Categorization). Finally, the information gathered in different categories was abstracted into results (see Table 1). The qualitative data software Atlas.ti 9 was used to assist in the open coding process and in the categorization of the codes in thematic groups.

**Table 1:** The three steps of the inductive content analysis.

Open coding	Categories	Abstraction
Channeling capacity; channeling online gambling demand toward an interesting offer; channeling and gambling harms; market share; channeling and gambling proceeds; consumer behavior	Limiting losses to offshore providers; Channeling is allowed;	Channeling strategy
Enforcement tool; gambling policy and blocking; market share without blocking measures; online gambling and blocking	Adequate blocking measures; Acceptability of blocking	Conditions of block offshore gambling without prohibiting online gambling
Licensing regimes and blocking; legitimacy of the monopolistic regime; offshore gambling and licensing regime	Purpose of the monopolistic regime; Challenging the current regime; Changing regime will not bring Veikkaus down	Shifting from a monopolistic to a licensing regime

## 4 Results

Despite various efforts to safeguard Finnish gambling offer against online offshore competition (Selin, 2019), Veikkaus’ online market share has been decreasing. During the period when the interviews were conducted, the business strategy of the state-owned Veikkaus relied of the principle of a ‘competitive monopoly’ (see Järvinen-Tassopoulos, 2022). The decreasing market share and the continuous loss of gambling profits to offshore providers had become a pressing matter. Channeling gambling demand without any proper measures to prevent offshore gambling seemed futile. The interviewees were simultaneously concerned with and aware of the pros and cons of channeling. Some of them were also well informed about how gambling, and channeling, was operated in other Nordic countries (such as Sweden and Norway).

### 4.1 Looking for an adequate channeling strategy

Both the representatives of Veikkaus and the public servants discussed at length the ideas of channeling gambling toward legal offer and curbing online gambling on unlicensed offshore websites. The representatives of Veikkaus saw channeling as a way to promote and develop the gambling company’s offer and to keep Finnish gamblers on Veikkaus’ website. The biggest issues for them were the loss of gambling proceeds to offshore providers and the number of Finnish gamblers playing on offshore websites.

“We should have a channeling strategy (in Finland) so that we (Veikkaus) could divert online gambling from offshore websites to Veikkaus’ website. Every year approximately 260 million Euros is lost (to offshore operators). Veikkaus’ gambling offer should be good enough so that customers would not play elsewhere.” (VE9, board of directors).

Previous studies have shown that gamblers choose a website according to awareness (e.g., the website is well-known, or the website has a good reputation), introductory deals and offers, pay out rates, customer protection, game fairness, and financial security (Zborowska et al., 2012; Gainsbury et al., 2018b). Within the EU, a state-owned gambling company is regulated otherwise than a private gambling company. To justify the significant market restriction of a monopolistic regime within the European single market, gambling proceeds must not be the main purpose of gambling operation. At the same time, a successful channeling strategy requires for the national gambling provider(s) to be interesting enough for players (*cf.* Pallesen et al., 2023).

Gambling regulators in Europe use a variety of measures to prevent offshore gambling providers from offering their products and services to gamblers (Hörnle et al., 2018; Schmidt-Kessen et al., 2019). Particularly, the use of website blocking as an enforcement measure has been popular: The measure is used by gambling regulators in more than half of all EU/EEA Member States (Schmidt-Kessen et al., 2019). As the blocking measures were not yet implemented in Finland during the time the interviews were conducted, the interviewees meant with ‘blocking’ either website or monetary transaction blocking. The representatives of Veikkaus considered blocking to be a worthwhile measure to be taken into account while searching for efficient means to limit unlicensed gambling offer in Finland.

“I can understand that many people believe that there are no proper measures (to regulate online gambling). Well, blocking is of course one measure that could be considered (in Finland). As in other European countries, the licensing system protects the gambling regime so that there are no free riders, and one way to deal with these free riders is to block them. Until now the measures used in the (Finnish) online market have increased (gambling) regulation instead of diminishing it. I am not talking about gambling limits, even though they mean well (to gamblers), but these limits have their own price tag. I mean that these gambling limits are not very useful, if the (Finnish) gamblers do not play (on Veikkaus’ website). I think this should be our common interest to bring them to our website.” (VE17, executive level).

In this excerpt, the interviewee engages the interviewer in a dialog for a common cause, which should be in their mind channeling Finnish gambling demand toward the national online market. Before the implementation of the channeling measures, such as payment blocking, most of the representatives of Veikkaus criticized the regulation of their gambling company as implementing online gambling limits was a costly task and the development of online products and services seemed to be impossible in the current regulatory framework.

Gambling regulators have essentially two options when it comes to blocking illegal gambling websites. They can block websites by targeting the server hosting the website, thus effectively removing the website from the Internet. This can be accomplished by take-down requests sent to the web hosting provider operating the server. Regulators can also block websites by preventing clients from accessing the website (Internet access blocking) (Schmidt-Kessen et al., 2019).

The next representative of Veikkaus acknowledges the issue with the offshore providers but understands that the regulation of unlicensed offer acquires high-level political decision-making:

“But then we should have a discussion on whether we should be able to limit the activity (of the unlicensed offshore companies), as they are legal operators in their own jurisdiction. Since they do not have a gambling license in Finland, we talk about blocking them. Is it blocking money transfers or IP addresses, well should the new government (of PM Sanna Marin) and the owner and perhaps even the members of Parliament discuss it?” (VE16, administrative board).

As previous studies have shown, different jurisdictions have tackled the issue of offshore gambling by different measures depending on how their legislation regulates online gambling (Gainsbury et al., 2018b; Egerer and Marionneau, 2023). According to the representatives of Veikkaus, the state-owned gambling company’s ability to compete with the offshore gambling companies was undermined by the regulators, which did not quite understand the consequences of the decreasing online market share. In addition to the failure of the channeling policy, the Finnish state could lose its interest in Veikkaus (Järvinen-Tassopoulos, 2022).

### 4.2 Retaining players on a website without prohibiting online gambling

In comparison to the discourse of the representatives of Veikkaus, the public servants had more heterogeneous views on the topic of channeling. Most of these interviewees voiced concern about the prevention of gambling harms. Here, one of the public servants explains channeling from a preventive perspective:

“If we want the Finnish gambling operator Veikkaus to offer games and we want Finns to play these games, then we need to think about consumer protection, crime prevention, and prevention of gambling harms. If we agree on these matters and it is politically outlined as such in this (monopolistic gambling) regime, then we need to find an appropriate gambling offer to channel online gambling to Veikkaus’ website. Our juridical answer is that according to the EU legislation, the purpose (of gambling operation) is preventing of gambling harms, and thus channeling can be allowed.” (PS3, regulation).

Offshore gambling offers have been depicted as more attractive and exciting to consumers than gambling products made available by legal gambling providers (Gainsbury et al., 2018a). Channeling is not only about the right kind of gambling products and services, but it is also about retaining the customers on the licensed and regulated gambling site:

“If the consumer were to act in a simple way, channeling would mean transferring gambling from one website to another. Then there would be no problem because the consumer would choose games that are less harmful. But what really happens is that consumer behavior can be changed with marketing and product development. What happens when Veikkaus creates new consumption? What will the consumers do when they discover for example online slot machines? Will they remain on the Veikkaus’ website? These are complicated issues that have not yet been studied enough.” (PS13, prevention of gambling harms).

While the interviewee (PS3) above talked about an ‘appropriate gambling offer’, the latter interviewee (PS13) questions the plans of the Finnish gambling provider to develop their gambling products and services. If the main purpose of the Finnish gambling policy is to prevent and limit gambling harms, then channeling online gambling toward the Veikkaus’ website cannot encourage consumers by advertising campaigns or by reminding gamblers about the ‘good causes’ to which gambling proceeds have been distributed in the Finnish welfare society (Van Den Bogaert and Cuyvers, 2011; Littler and Järvinen-Tassopoulos, 2018; Marionneau and Kankainen, 2018).

Some of the public servants saw channeling as a means to maintain the current monopolistic regime in Finland. Whereas most other Nordic and European countries have chosen to open their online gambling market to licensed gambling providers (e.g., Cisneros Örnberg and Hettne, 2018; Forsström and Cisneros Örnberg, 2019), Finnish regulators were still looking for measures to limit offshore gambling offer within the monopolistic regime:

“We are currently examining how to block offshore gambling with technical measures. Preventing is a technical issue, but also a juridical issue. Online gambling is not prohibited by the (Finnish) Penal Code. We (the regulators) have contemplated on legal issues and sometimes we have done so even with Veikkaus. The problem is not the gambler. If we think about offshore provision, which is not regulated by the (Finnish) Lotteries Act, this is precisely the issue we would like to tackle. Even the National Police Board is prepared to examine options to block (offshore operation). However, this online environment without visible borders, it is a challenge for regulators.” (PS3, regulation).

Starting in May 2018, the Finnish Ministry of the Interior prepared to amend the Lotteries Act (1047/2001). One of the aims of this preparative phase was to strengthen the channeling capacity of Veikkaus. In addition, adequate technical measures were investigated to block offshore gambling offer (Rydman and Tukia, 2019). This ensuing report (Rydman and Tukia, 2019) dealt with blocking access to websites and blocking monetary transactions between online gambling operators and gamblers.

One of the public servants anticipated how hard it would be for Finnish gamblers to accept blocking measures preventing their access to offshore websites:

“Finns are used to gambling online, so it would not fit to their mindsets if Internet were to be blocked like in North Korea, or offshore gambling were to be prohibited by law. The operation of Veikkaus, its regulation, the (Finnish) gambling legislation, and offshore operation take place in the same (online) environment. We do not have a protected system (in Finland). In the Netherlands, online gambling was prohibited, and there was no Dutch operator to online games.” (PS12, regulation).

The Dutch case was particularly interesting to the Finnish regulators in 2018 and 2019. The interviewee (PS12) seems to depict the Dutch gambling regime more protected from the offshore gambling offer than the Finnish gambling regime. However, online sports betting and horserace betting were available through an e-commerce exception in the Netherlands (Littler and Järvinen-Tassopoulos, 2018). Also, even though payment transactions could not be blocked under the Dutch Betting and Gaming Act, payment blocking was based on “voluntary co-operation” by the service providers (Hörnle et al., 2018, p. 59; Rydman and Tukia, 2019).

When the interviews were conducted, offshore gambling provision challenged the Finnish gambling policy. The Finnish gambling regime had become unable to divert online gambling demand toward Veikkaus’ offer. While the representatives of Veikkaus were more interested in allowing Veikkaus to evolve and to develop new products and services, the Finnish public servants were more concerned about the prevention of gambling harms and consumer protection. In the interviews, channeling was described as an important policy tool to curb offshore provision, but without a firm political will and the creation of a regulatory framework to block offshore operation and marketing, channeling would be more of an ideal type of political strategy than a robust and realistic one.

### 4.3 Toward a licensing system?

In 2018, the total worth of the Finnish gambling market amounted approximately to € 2,050 million (Veikkaus’ gross gaming revenue (GGR) combined with the estimate of H2 Gambling Capital of the offshore gambling provision) and Veikkaus’ share of this market was 89 percent (Veikkaus, 2018). Veikkaus updated its business strategy between the end of 2019 and the beginning of 2020 due to the public backlash concerning its marketing and the volume of electronic gambling machines outside casinos. Instead of keeping competitiveness among its core strategical values, new core themes were responsibility, channeling capacity, and new businesses (e.g., B2B operations) (Veikkaus, 2019).

All the interviewees were asked how they would define the purpose of the monopolistic gambling regime in Finland. Most of the public servants referred to the Finnish Lotteries Act in their answers:

“Well, (the purpose) is written in the gambling legislation. It is seen (in Finland) that the monopolistic regime is a better way to prevent gambling harms than any other regime. (Veikkaus) is steered by the state that is the owner. From my point of view, steering is a procedure, and it also includes the work of the Harm Assessment Group.” (PS13, prevention of gambling harms).

According to the Lotteries Act, the prevention of gambling harms is the legal justification of the Finnish monopolistic regime alongside consumer protection and crime prevention. In the excerpt above, the public servant (PS13) talks about steering, which according to them, does not simply take the form of ownership steering. The Harm Assessment Group is assigned by the Finnish Ministry of Social Affairs and Health, and it issues assessments and reports on gambling policy and operation. Thus, by its work, it steers gambling policy and operation from a preventive perspective.

Since 2013, when the EC stated that Finnish gambling legislation complies with EU law, Finnish regulators have increasingly strengthened the different responsibility measures to limit and prevent gambling harms. In December 2017, new loss limits for fast-paced online games and for money transfers to online gaming account were established, gamblers could exclude themselves from all online gambling, and they could set a time counter to keep track of time spent gambling online (Järvinen-Tassopoulos, 2018). During the COVID-19 pandemic, the loss limits in online gambling were lowered (Järvinen-Tassopoulos et al., 2020).

Another public servant, while discussing the purpose of the monopolistic gambling regime, expressed their point of view of the reasons precluding Finland from opening its online gambling market to new providers:

“I think that no one has dared to challenge the monopolistic gambling regime. The regime is like the holy truth because the benefits are huge and the regime finances (with the gambling proceeds) so many activities. Those beneficiaries are very influential. If the state were to end the monopolistic regime, it would have to assure the beneficiaries that their status would not weaken. Try to weaken it and you will see how hard it is.” (PS1, prevention of gambling harms).

This excerpt represents ‘beneficiaries’ of gambling proceeds (e.g., non-governmental organizations) as prominent stakeholders intent on maintaining their funding. It also implies that the Finnish civil society is dependent of the gambling proceeds and thus of the monopolistic regime. At the same time, previous studies have shown that accepting money from the gambling industry may be ethically dubious and even problematic for those receiving it (Marionneau and Kankainen, 2018). In other words, maintaining such a transactional relationship with various beneficiaries transforms the role of the Finnish state from policy maker to revenue collector (Adams, 2008; Orford, 2011).

While most of the interviewees agreed on the purpose of the monopolistic regime, some of them commented on the possibility of establishing a licensing regime in Finland. This possibility was generally discussed consequently to Veikkaus’ decreasing online market share. According to these interviewees, the payout rates, license fees, the gambling proceeds, and even the customer experience were on a different level in a licensing regime. Opening the online gambling market can also prove to be risky to the state as well as to the gamblers:

“I wish that the Finnish gambling regime would not change into something else, because the alternative has two negative consequences. First, dozens of gambling operators compete (in a licensing regime). The competition will get harder and the means to compete will be, how to say it properly, more efficient. So, other (offshore) operators will have means to compete that Veikkaus does not have, and this will lead to an increase in online gambling (among Finnish gamblers). Simultaneously, there is a strong probability that this (competition) will lead to a decrease of gambling proceeds. (…) Secondly, when we look at opened gambling markets, the tax on the GGR is between 10 and 20 percent. This means that gambling (in Finland) should triple (in a licensing regime) so that the (Finnish) state would collect the same amount of gambling proceeds (as in a monopolistic regime). Finns already gamble a lot compared to other nationalities. If they were to gamble twice as much, well no one wants to see that happen in Finland. From the perspective of Veikkaus, the company will continue to operate regardless of the gambling regime.” (VE14, executive level).

The interviewee depicts the licensed market almost as chaotic as competition with offshore providers that cannot be regulated or taxed (*cf.* the ‘wild west’ metaphor in Cisneros Örnberg and Hettne, 2018). The excerpt echoes the need to maintain the same level of gambling proceeds in another gambling regime but recognizes that increased online gambling may increase the prevalence of gambling harms. When Sweden decided to re-regulate online gambling under a licensing regime, it imposed an 18 percent tax on GGR on the newly licensed providers (Cisneros Örnberg and Hettne, 2018). Then again, in France the tax level of gambling is 33.7 on GGR for online sports betting and 1.8 percent on turnover for online table games (Vila, 2020).

The Swedish case interested many interviewees as Sweden opened its online market to former offshore providers during the time the interviews were conducted. Here, a public servant challenges the Swedish licensing process of online gambling products:

“It is quite useless to compare Finland and Sweden because the latter has adopted a licensing regime. Sweden has maintained monopoly in products such as lottery, which is quite odd. I mean, the products that are the least harmful are left out (from the licensed game selection) because their return potential is the biggest and the (Swedish) state wants to keep these products. How can EU accept this? The (Swedish) monopoly should include products with the most harmful features. Either way, Sweden has moved on to a licensing regime in which various gambling companies operate legally.” (PS2, regulation).

A licensing regime can employ different measures to prevent gambling harms. Identification, monetary and temporal limits, and self-exclusion are measures that are also used in monopolistic gambling regimes. One of the more specific measures is called ‘duty of care’ that requires gambling companies to alert or contact gamblers when their gambling behavior becomes risky and to provide them with information about help and where to find it (Forsström and Cisneros Örnberg, 2019). In addition, maintaining blacklists of unlicensed offshore websites, restricting transactions to offshore websites, limiting advertising, and the use of landing pages to inform the gamblers are channeling measures in a licensing regime (Gainsbury et al., 2018a; Schmidt-Kessen et al., 2019). Prevention of gambling harms online starts with gambling products and services, but it needs adequate gambling policies and robust regulation of online gambling operation to succeed.

When the interviews were conducted, the future of the Finnish online market seemed uncertain. Nevertheless, the purpose of the monopolistic regime was clear to the interviewees. Some of the interviewees were ready to talk about an alternative gambling regime but their vision of it was based on their knowledge of the Swedish and Dutch cases. Even though channeling does not depend on a specific gambling regime, it is a strategic tool used by governments to divert gambling demand from unlicensed offshore offer to licensed offer (Borch, 2022). In Finland, channeling has been more a policy rationale until January 2023, when the amendment of the Lotteries Act (1284/2021) took effect and prohibited unlicensed gambling providers to operate and advertise in Finland by threating them with sanctions and fines. This study has shown that channeling becomes an effective strategic tool only then when proper measures are used to limit the access of offshore providers to the national online gambling market and to prevent gambling harms.

## 5 Discussion

The results of this study have shown that the rising importance of offshore gambling operation can have profound implications for the regulation of gambling at a jurisdictional level. Since 2018, the loss of market share to offshore providers has led Finnish stakeholders to emphasize the channeling policy. The channeling objective was used to justify varying policy approaches, including amending the Finnish Lotteries Act and by investigating for appropriate measures to block offshore provision (see Rydman and Tukia, 2019). In addition, some of the interviewees anticipated the possibility to move from a monopolistic gambling regime to a licensing regime. However, during the time of the interviews, no one seemed to be ready for this change. Particularly, many interviewees were aware of recent developments in Sweden where unsuccessful attempts to channel gambling demand to the monopoly eventually led to the introduction of a licensing regime in 2019. This change of regime was preceded by the decreasing of the monopolistic share of the Swedish online gambling market to 47 percent (Selkee et al., 2022). The results show that as the share of online gambling increases, channeling can become the guiding principle of national gambling regulation.

Previous research has suggested that channeling policies are justified in terms of higher levels of consumer protection, fewer criminal activities, and surplus of profits (*cf.*, Van Den Bogaert and Cuyvers, 2011; Borch, 2022; Selkee et al., 2022). Our results partly support these findings. In our study, channeling was seen particularly as a tool of preventing gambling harms (the socio-economic hypothesis according to Nadeau et al., 2014). The representatives of Veikkaus believed that the online gambling offer of the state-owned gambling company was less harmful (and thus more responsible) than those of offshore competitors. For example, Veikkaus did not offer welcome bonuses or free spins to consumers (see Gainsbury et al., 2018b). The surplus of profits was also mentioned as a characteristic of the Finnish monopolistic gambling regime. Finally, none of our interviewees discussed criminal activity. Some representatives of Veikkaus did criticize offshore operators for being untrustworthy, but more systemic criminality, such as money laundering or match fixing, was not mentioned.

Approaches to gambling regulation vary across times and policy goals. In order to understand the regulatory and political circumstances related to the channeling approach, that also led the new Finnish government to decide to change its approach to the licensing of online gambling providers, we adapted Kingma’s (2008) theory on gambling regulation to our study. By creating different prototypes of regulatory models, Kingma (2008) has shown how the liberalization and the virtualization of gambling operation has changed the regulation of gambling in the Netherlands. In Kingma’s theory, the ‘alibi model’ refers to the legalization of gambling to avoid illegal markets, and restricted offer by discouraging private pursuit of profit. Under this model, gambling proceeds are allocated to ‘good causes’ (Kingma, 2008; Chambers, 2011).

Instead of using the ‘risk model’, which represents regulatory change from the ‘alibi model’ in the Dutch case, we propose a new model representing the Finnish online gambling policy starting from 2018 (see Table 2). Kingma’s (2008) ‘risk model’ offers a ‘liberal political approach’ (Chambers, 2011) to gambling, which is seen as a commercial entertainment. In the ‘risk model’, the economic importance of the gambling sector is acknowledged, and the controlled gambling market is preventing risks such as addiction and crime. Our ‘dam model’ captures gambling policy that aims to channel online gambling to the regulated offer, and to actively prevent offer from elsewhere. The ‘alibi model’ has illustrated the Finnish online gambling system well (Littler and Järvinen-Tassopoulos, 2018), but a shift in policy took place around 2018, spearheaded by the channeling objective.

**Table 2:** Models of online gambling regulation in Finland [based on Kingma (2008) and Littler and Järvinen-Tassopoulos (2018)].

	‘Alibi model’	‘Dam model’
Time frame	2000s onwards	2018 onwards
Moral meaning of online gambling	It is a potentially harmful activity	Offshore gambling is harmful for the consumer and the state
Political strategy	Status quo	Channeling online gambling toward regulated gambling
Rationale for gambling law	Prevention of gambling harms and crime	Prevention of gambling harms and crime
Proceeds	Good causes	Toward state budget
Central concern	Competition from unlicensed cross-border gambling operators, loss of gambling proceeds	Protecting the online market share, blocking offshore gambling, competitiveness of Veikkaus
Exploitation	Monopoly system	Monopoly system with a less regulated state-owned gambling company
Controlling institutions	Legislation and gambling administration	Amendment of the Lotteries Act
Ideal state	The welfare state	Competition society

Table 2 presents how the channeling approach, or ‘dam model’ differs from the alibi model in the Finnish context of online gambling regulation. In the 2000s, online gambling was considered as a potentially harmful activity, best regulated in a monopolistic regime. Competition from unlicensed online gambling operators existed to some extent, but policy makers were more concerned with justifying the monopolistic gambling regime to the European Commission stressing the importance of the prevention of gambling harms (Littler and Järvinen-Tassopoulos, 2018). The ‘alibi model’ indicated a “social democratic view” (Myllymaa, 2017, p. 233) of gambling, meaning that gambling was considered as an exceptional activity that does not produce any kind of added value necessary for increased economic growth and value. The ideal state is the Finnish welfare state in which welfare is provided by the public sector (state and municipalities), the private sector (market), civil society (third sector organizations, nongovernmental organizations) and family (Pessi and Grönlund, 2011).

Despite the many efforts to amend the Finnish Lotteries Act in the 2000s and 2010s (Selkee et al., 2022), the main political strategy in Finland was to maintain and strengthen the monopolistic gambling regime in order to limit the competition from inside (between three national gambling companies) and outside (between Veikkaus and unlicensed competitors). This strategy has influenced the moral meaning of online gambling and the rationale for gambling law in Finland. In addition, it seems that this monopolistic regime, as well as its strong regulatory model, has suited well the ideal of the welfare state in which gambling proceeds become ‘good causes’. Yet Hellman and Alanko (2021) have pointed out that using gambling proceeds to fund the third sector is problematic, as the public sector and third sector can have conflicting roles (e.g., nongovernmental organizations compensate the lack of services or they provide alike services), and the third sector’s work is counteracted by the gambling activity that funds the services (e.g., large part of the gambling proceeds come from individuals with gambling problems) (*cf.* Table 1, Hellman and Alanko, 2021, p. 96).

The amendment of the Finnish Lotteries Act started in May 2018 and its aims included the strengthening of Veikkaus’ channeling capacity and the blocking of offshore provision (Rydman and Tukia, 2019). The regulation of online gambling has therefore shifted to a new model (the ‘dam model’) that aims to improve channeling measures and protect the Finnish online market from unlicensed gambling providers. The moral meaning of online gambling has changed since 2018: As also highlighted by the interviewees in our study, offshore gambling was seen as more harmful to Finnish gamblers than gambling on a national, regulated website. To ensure the competitivity of Veikkaus in the face of offshore competition, as also highlighted in the company’s business strategy in 2018, the Finnish society (represented by the public servants in this study) would need to create new solutions to safeguard the monopolistic regime and to consolidate the channeling policy. The latter matter would succeed better if the state-owned gambling company was less regulated and granted the same means than offshore companies to entice customers (e.g., welcoming bonuses) (*cf.* Järvinen-Tassopoulos, 2022).

The ideal type of ‘competition society’ (see Heiskala and Luhtakallio, 2006; Rainio-Niemi, 2015) refers to the impact of globalization on the Finnish society and to the radical changes on economic (competitiveness), technological (development and spreading of information technology), institutional (pressures to renew regulation), organizational (spreading of networks), productive (virtualization of products, accentuation of competition and the quality of demand) and public policy (competitiveness and growth) levels in Finland (Heiskala and Luhtakallio, 2006; Rainio-Niemi, 2015). In Table 2, the term depicts the virtualization of gambling which has required a new political stand from the Finnish society regarding online competition and the evolution of the online gambling market. Internet is the metaphor of a globalized world in which a ‘corporatist political approach’ (Chambers, 2011), such as the ‘alibi model’, may not respond anymore to the continuous competition that rules the global online gambling market.

As the gambling proceeds will be transferred to the state budget without earmarking from 2024, it can be anticipated that this change will guarantee the consistency of the gambling regime with the values of the Nordic welfare state model (Hellman and Alanko, 2021). Nevertheless, competitiveness has been the keyword for the neoliberal governance and from the perspective of the Nordic welfare state, this has meant guaranteeing optimal conditions for the accumulation of capital and for the maximal utilization of the work force (Saarinen et al., 2014). This in mind, it is possible to presume that the ‘dam model’ will be a temporary model that will be replaced by a more adequate regulatory model when the current gambling regime will end.

The results have important implications on policy. When channeling becomes the guiding principle of gambling regulation, this can overshadow other regulatory concerns. Channeling suggests that regulated or domestic offer of gambling is a ‘safer’ or ‘better’ option although all forms of gambling, regulated or offshore, can lead to significant harms. By 2026, the Finnish gambling market is set to be partially opened for licensed online gambling providers. Nevertheless, the channeling objective is likely to remain a guiding principle. In other European countries that have recently adopted a licensing system, such as the Netherlands or Sweden, channeling rates to the regulated system remain a central policy concern and indicator of systemic success. Going forward, the channeling objective in Finland may become increasingly tied to the socio-sanitary hypothesis (Nadeau et al., 2014) according to which a wider selection of gambling operators may increase total consumption and harms. However, as the results have suggested, a monopoly is also not immune to competition. Rather than focusing on channeling the consumption of gamblers to regulated offers, future gambling policies should perhaps focus on channeling the consumption away from gambling altogether, or at least, away from the most harmful products.

The current study has some limitations. Our data consist of key informant interviews involved in the regulation and provision of gambling. Their perspectives on gambling can be influenced by their position and the ‘profile effect’. Furthermore, the study has been limited to the case of Finland to describe a shift in regulatory models from a ‘alibi model’ toward a new ‘dam model’. The Finnish case is unlikely to be fully representative of other jurisdictions in Europe or elsewhere. Notably, the ‘risk model’ has largely been missing in the Finnish context. Channeling has different meanings across contexts, and these have translated into varying modes of implementation (*cf.* Van den Bogaert and Cuyvers, 2011; Borch, 2022; Järvinen-Tassopoulos, 2022; Selkee et al., 2022). Also, the Finnish monopolistic gambling regime has been historically related to the funding of ‘good causes’ with gambling proceeds (*cf.* ‘common good’, ‘public interest’), which is not the case in jurisdictions where these proceeds are part of the state budget (Järvinen-Tassopoulos and Eräsaari, 2018; Sulkunen, 2018).

More research from other contexts would be necessary to ascertain whether a shift to the ‘dam model’ of regulation applies elsewhere and whether its underlying principles and rationales differ elsewhere. Furthermore, regulatory models are ideal types rather than clearly defined or mutually exclusive practical applications. As also noted by Kingma (2008), various regulatory principles can also coexist or compete. In some contexts, and particularly those in which the gambling markets have been opened to permissive licensing regimes, this might mean that the ‘dam model’ takes on more traits from a ‘neo-liberal view’ (Myllymaa, 2017, p. 233) according to which gambling is just another form of business activity and gamblers spend their money on what yields them most ‘consumer welfare’ or ‘consumer surplus’.

## 6 Conclusion

Channeling is a specific regulatory tool, the purpose of which is to direct online gambling from unlicensed offshore gambling toward the national online gambling market in order to prevent gambling harms online, and to protect the online market share of national or state-owned gambling companies. As a regulatory tool, channeling is not dependent on a specific legal system or gambling regime. In addition, channeling is composed of various policy measures, including making national offer more attractive, or outside offer less attractive. Following the channeling principle, encapsulated in the ‘dam model’, payment blocking measures were implemented in Finland in the beginning of 2023. However, and as our study has also shown, the channeling principle alone does not completely solve the issue of offshore gambling provision without additional measures. Furthermore, an overly emphasized focus on channeling can overshadow problems in the national market.

National stakeholders hold important information on the evolution of the online gambling market, on the legislative development in the jurisdiction, and on the regulatory challenges caused by online gambling. This study indicates that legislative and regulatory changes are lengthy political processes, which are defined by agenda setting, decision-making, and implementation (Zahariadis, 2014). Further studies are needed to document the full policy process related to the passage from one gambling regime to another and the shifts to and from the ‘dam model’ in Finland and elsewhere.

## Data availability statement

The raw data supporting the conclusions of this article will be made available by the authors, without undue reservation.

## Ethics statement

Ethical approval was not required for the studies involving humans because the executive team of Veikkaus granted permission to interview its employees for research purposes. The public servants accepted individually to be interviewed for research purposes. The studies were conducted in accordance with the local legislation and institutional requirements. The participants provided their written informed consent to participate in this study.

## Author contributions

JJ-T: Conceptualization, Formal analysis, Funding acquisition, Investigation, Methodology, Software, Writing – original draft, Writing – review & editing. VM: Conceptualization, Funding acquisition, Investigation, Writing – original draft, Writing – review & editing. ME: Conceptualization, Funding acquisition, Writing – review & editing.

## References

[ref1] Abels G. Behrens M. (2009). “Interviewing experts in political science: a reflection on gender and policy effects based on secondary analysis” in Interviewing experts. eds. Bogner A. Littig B. Menz W. (Hampshire: Palgrave Macmillan), 138–156.

[ref2] Adams P. J. (2008). Gambling, freedom and democracy. London: Routledge.

[ref3] Banks J. (2014). Online gambling and crime: causes, controls and controversies. London: Routledge

[ref4] Banks J. (2017). Gambling, crime and society. London: Palgrave Macmillan.

[ref5] Borch A. (2022). “Channeling gambling: the case of Norway” in The global gambling industry. eds. Nikkinen J. Marionneau V. Egerer M. (Wiesbaden: Springer), 235–254.

[ref6] Chambers K. G. E. (2011). Gambling for profit. Lotteries, gaming machines, and casinos in cross-national focus. Toronto: University of Toronto Press.

[ref7] Cisneros Örnberg J. Hettne J. (2018). “The future Swedish gambling market: challengers in law and public policies” in Gambling policies in European welfare states. Current challenges and future prospects. eds. Egerer M. Marionneau V. Nikkinen J. (Cham: Palgrave Macmillan), 197–216.

[ref8] Costes J.-M. Kairouz S. Eroukmanoff V. Monson E. (2016). Gambling patterns and problems of gamblers on licensed and unlicensed sites in France. J. Gambl. Stud. 32, 79–91. doi: 10.1007/s10899-015-9541-2, PMID: 25862019

[ref9] Egerer M. Marionneau V. (2023). Blocking measures against offshore online gambling: a systematic review. Int. Gambl. Stud., 1–17. doi: 10.1080/14459795.2023.2190372

[ref10] Elo S. Kyngäs H. (2008). The qualitative content analysis process. J. Adv. Nurs. 62, 107–115. doi: 10.1111/j.1365-2648.2007.04569.x 18352969

[ref11] Forsström D. Cisneros Örnberg J. (2019). Responsible gambling in practice: a case study of views and practices of Swedish oriented gambling companies. Nordic Stud. Alcohol Drugs 36, 91–107. doi: 10.1177/1455072518802492, PMID: 32934553 PMC7434122

[ref12] Gainsbury S. M. Abarbanel B. Blaszczynski A. (2018a). Factors influencing internet gamblers’ use of offshore online gambling sites: policy implications. Policy Internet 11, 235–253. doi: 10.1002/poi3.182

[ref13] Gainsbury S. M. Russell A. M. T. Hing N. Blaszczynski A. (2018b). Consumer engagement with and perceptions of offshore online gambling sites. New Media Soc. 20, 2990–3010. doi: 10.1177/1461444817738783

[ref14] Gläser J. Laudel G. (2009). “On interviewing “good” and “bad” experts” in Interviewing Experts. eds. Bogner A. Littig B. Menz W. (Hampshire: Palgrave Macmillan), 117–137.

[ref15] Growth Marketing Report. (2023). Online Gambling Market. Available at: https://growthmarketreports.com/report/online-gambling-market-global-industry-analysis

[ref16] H2 Gambling Capital. (2021). An overview of Finland’s gambling market. Available at: https://www.egba.eu/uploads/2021/09/H2-EGBA-Presentation-September-2021.pdf

[ref17] Heiskala R. Luhtakallio E. (2006). “Johdanto: Suunnittelutaloudesta kilpailukyky-yhteiskuntaan” in Introduction: From planned economy to competition society, in Uusi jako. Miten Suomesta tuli kilpailukyky-yhteiskunta? [New Deal. How did Finland become a competition society?]. eds. Heiskala R. Luhtakallio E. (Helsinki: Gaudeamus), 7–13.

[ref18] Hellman M. Alanko A. (2021). “System level resilience: the role of welfare state accountability” in The political Analyst’s field guide to Finland. eds. Ilmonen K. Moilanen P. (Jyväskylä: University of Jyväskylä, Finland), 84–102.

[ref19] Hörnle J. Littler A. Tyson G. Padumadasa E. Schmidt-Kessen J. M. Ibosiola D. I. (2018). Evaluation of regulatory tools for enforcing online gambling rules and Channelling demand Towrds controlled offers. Final report. Brussels: European Commission.

[ref20] Järvinen-Tassopoulos J. (2018). State of play 2017: A review of gambling in Finland. Helsinki: National Institute for Health and Welfare.

[ref21] Järvinen-Tassopoulos J. (2022). “State-owned gambling operation in a global competitive environment” in The global gambling industry. eds. Nikkinen J. Marionneau V. Egerer M. (Wiesbaden: Springer), 27–40.

[ref22] Järvinen-Tassopoulos J. Eräsaari R. (2018). “Conceptions of the common good” in Gambling policies in European welfare states. Current challenges and future prospects. eds. Egerer M. Marionneau V. Nikkinen J. (Cham: Springer), 259–274.

[ref23] Järvinen-Tassopoulos J. Marionneau V. Lerkkanen T. (2020). Rahapelaaminen koronapandemian aikana. Kokemuksia riskeistä ja muutoksista [gambling during the COVID-10 pandemic. Experiences of risks and changes]. Tiede Edistys 4, 386–406. doi: 10.51809/te.109680

[ref24] Kingma S. F. (2008). The liberalization and the (re)regulation of Dutch gambling markets: national consequences of the changing European context. Regulat. Governance 2, 445–458. doi: 10.1111/j.1748-5991.2008.00045.x

[ref25] Littler A. (2011). Member states versus the European Union: The regulation of gambling. Leiden: Martinus Nijhoff Publishers.

[ref26] Littler A. Järvinen-Tassopoulos J. (2018). Online gambling, regulation, and risks: a comparison of gambling policies in Finland and the Netherlands. J. Law Social Policy 30, 100–126. doi: 10.60082/0829-3929.1324

[ref27] Lycka M. (2014). Taxation of gambling in Europe—barrier to entry into new markets? Gaming Law Rev. Econ. 18, 288–296. doi: 10.1089/glre.2014.1835

[ref28] Marionneau V. Kankainen V. (2018). Beneficiaries of gambling and moral disengagement. Int. J. Sociol. Soc. Policy 38, 578–591. doi: 10.1108/IJSSP-01-2018-0005

[ref29] Miettinen S. (2022). “Which ends justify the means? Justifying National Restrictions to the free movement of gambling Services in the European Union” in The global gambling industry: Structures, tactics, and networks of impact. eds. Nikkinen J. Marionneau V. Egerer M. (Wiesbaden: Springer), 219–233.

[ref30] Myllymaa A. (2017). The political economy of online gambling in the European Union. Doctoral dissertation. Faculty of Social Sciences 134. Helsinki: University of Helsinki.

[ref31] Nadeau L. Dufour M. Guay R. Kairouz S. Ménard J. M. Paradis C. (2014). Online gambling: When the reality of the virtual catches up with us. Montréal: Working Group on Online Gambling.

[ref32] Orford J. (2011). An unsafe bet? The dangerous rise of gambling and the debate we should be having. Chichester: Wiley-Blackwell.

[ref33] Pallesen S. Mentzoni R. A. Syvertsen A. Hellumbråten Kristensen J. Erevik E. K. Morken A. M. (2023). Omfang av penge- og dataspillproblemer i Norge 2022 [prevalence of gambling and gaming in Norway 2022]. Bergen: University of Bergen.

[ref34] Pessi A. B. Grönlund H. (2011). The place of the church: public sector of civil society? Welfare provision of the evangelical Lutheran Church of Finland. J Church State 54, 353–374. doi: 10.1093/jcs/csr087

[ref600] Planzer S. (2014). Empirical views on European gambling law and addiction. Cham: Springer.

[ref35] Rainio-Niemi J. (2015). “A Nordic paradox of openness and consensus? The case of Finland” in The paradox of openness, transparency and participation in Nordic cultures of consensus. eds. Götz N. Marklund C., vol. 126 (Leiden: International Studies in Sociology and Social Anthropology), 29–47.

[ref36] Rolando S. Scavarda A. (2018). “Italian gambling regulation: justifications and counter-arguments” in Gambling policies in European welfare states. Current challenges and future prospects. eds. Egerer M. Marionneau V. Nikkinen J. (Cham: Springer), 37–57.

[ref37] Rydman E. Tukia J. (2019) Rahapelilainsäädäntöä koskeva esiselvitys [Pilot study regarding gambling legislation]. Sisäministeriön julkaisuja 2019: 25. Helsinki: Sisäministeriö.

[ref38] Saarinen A. Salmenniemi S. Keränen H. (2014). Hyvinvointivaltiosta hyvinvoivaan valtioon. Hyvinvointi ja kansalaisuus suomalaisessa poliittisessa diskurssissa [from welfare state to welfare of the state. Welfare and citizenship in Finnish political discourse]. Yhteiskuntapolitiikka 79, 605–618.

[ref39] Sailas H. Brax T. Matilainen R. Alkio M. (2023). Esiselvitys rahapelijärjestelmän vaihtoehdoista [preliminary study on alternative models for the gambling system]. Helsinki: Sisäministeriö.

[ref40] Schmidt-Kessen M. J. Hörnle J. Littler A. (2019). Preventing risks from illegal online gambling using effective legal design on landing pages. Copenhagen Bus. School Law Res. Paper, 19–36. doi: 10.2139/ssrn.3474296

[ref41] Selin J. (2019). National gambling policies and the containment of the EU’s politico-legal influence. Nordic Stud. Alcohol Drugs 36, 77–90. doi: 10.1177/1455072519835703, PMID: 32934552 PMC7434120

[ref42] Selkee M. Selin J. Raisamo S. Lintonen T. (2022). Rahapelaamisen kanavointi. Käsitteen käyttö ja sen merkitykset eri Pohjoismaissa. [channeling of gambling. The use of the concept and its definitions in the Nordic countries]. Yhteiskuntapolitiikka 87, 271–284.

[ref43] Spapens T. Littler A. Fijnaut C. (2008). Crime, addiction and the regulation of gambling. Leiden: Martinus Nijhoff Publishers.

[ref44] Sulkunen P. (2018). “The public interest approach to gambling policy and research” in Gambling policies in European welfare states. Current challenges and future welfare states. eds. Egerer M. Marionneau V. Nikkinen J. (Cham: Springer), 275–296.

[ref45] Sulkunen P. Berret S. Marionneau V. Nikkinen J. (2022). Commercial gambling and the surplus for society: a comparative analysis of European companies. Crit. Gambl. Stud. 3, 96–109. doi: 10.29173/cgs92

[ref46] Van Den Bogaert S. Cuyvers A. (2011). Money for nothing: the case law of the EU court of justice on the regulation of gambling. Common Market Law Rev. 48, 1175–1213. doi: 10.54648/COLA2011046

[ref47] Veikkaus. (2018). Corporate governance. Available at: https://cms.veikkaus.fi/site/binaries/content/assets/dokumentit/vuosikertomus/2018/veikkaus_corporate-governance_2018.pdf

[ref48] Veikkaus. (2019). Annual and CSR report. Available at: https://cms.veikkaus.fi/site/binaries/content/assets/dokumentit/vuosikertomus/2019/veikkaus_annual_csr_report_2019_200520.pdf

[ref50] Vila J.-B. (2020). Le droit de la régulation des jeux d’argent [Regulatory law in gambling]. Paris La Défense: LGDJ Lextenso.

[ref51] Zahariadis N. (2014). “Ambiguity and multiple streams” in Theories of the policy process. eds. Sabatier P. Weible C. (Boulder: Westview Press), 25–58.

[ref52] Zborowska N. Kingma S. F. Brear P. (2012). “Regulation and reputation. The Gibraltar approach” in The international handbook of internet gambling. eds. Williams R. J. Wood R. T. Park J. (London: Routledge), 84–100.

